# The high tolerance to aluminium in crucian carp (*Carassius carassius*) is associated with its ability to avoid hypoxia

**DOI:** 10.1371/journal.pone.0179519

**Published:** 2017-06-23

**Authors:** Antonio B. S. Poléo, Joachim Schjolden, Jørgen Sørensen, Göran E. Nilsson

**Affiliations:** 1 Inland Norway University of Applied Sciences, Campus Evenstad, Norway; 2 The Norwegian National Authority for Investigation and Prosecution of Economic and Environmental Crime, Norway; 3 Fresenius Kabi Norge AS, Norway; 4 Department of Biosciences, University of Oslo, Norway; VIT University, INDIA

## Abstract

It is well known that aluminium is the principle toxicant killing fish in acidified freshwater systems, and it has been shown that crucian carp (*Carassius carassius*) can survive exposures to aqueous aluminium levels toxic to most other freshwater fish species. The crucian carp has a remarkable ability to survive anoxic conditions, and the aim of the present study was to reveal if the tolerance to aluminium can be associated with the ability to survive prolonged anoxia. Crucian carps were exposed to either acidic Al-rich water (pH 5.8; 960 μg Al/l), acidic Al-poor water (pH 5.8; 50 μg Al/l) or untreated control water (pH 6.5; 50 μg Al/l). Blood, muscle and gill samples were collected from exposed fish, and closed respirometry was performed to measure critical O_2_-tension an normoxic O_2_-consumption. The results show an increased gill surface area in Al-exposed fish, while the critical O_2_-tension did not change. The normoxic O_2_-consumption was lower in Al-exposed fish and might be due to a reduced metabolic rate. The results suggest that crucian carp exposed to aluminium do not become hypoxic, since haematocrit, plasma lactate and blood ethanol did not differ from that of control fish after 14 days of exposure. We also observed an initial loss of plasma chloride and sodium, followed by a stabilisation of these ions at a lower level than in control fish. The decrease in plasma ions caused a transient increase in haematocrit and water content in muscle tissue, returning to control levels when the ion concentrations stabilised, suggesting that the water balance was restored. We conclude that the high tolerance to aluminium in crucian carp is associated with its ability to avoid hypoxia as well as an ability to counteract a continuous loss of plasma ions.

## Introduction

The potential for aluminium toxicity in freshwater organisms, especially fish, has for a long time been of major concern in the management of the aquatic environments affected by acid rain in the northern hemisphere. Increased concentrations of aqueous aluminium are confirmed to be the most important effect caused by this kind of freshwater acidification, and the relationship between aqueous aluminium and fish toxicity is quite extensively documented [1–3]. Accordingly, aqueous aluminium is now considered to be the principle toxicant killing fish in acidified waters [3–4]. Acid mine drainage is another common source of aluminium mobilisation from the edaphic to the aquatic environment [5–6], and which has been associated with fish toxicity [7].

It is well documented that Al-toxicity in various aquatic biota depends on changes in water physico-chemical conditions, rather than the presence or the concentration of a certain aluminium species [1, 3, 8–11]. Accordingly, it has been demonstrated that a non-steady state Al-chemistry often predominate in natural environments [12–15], in which monomeric Al-forms are transformed to polymeric Al-forms, may dictate the Al-toxicity [16]. When Al-solubility decreases as pH is elevated in an acidic water body, the toxicity to fish increases because aluminium starts to polymerise, and subsequently accumulates on the gill surface [10, 16]. The primary effect of Al-polymerisation and precipitation or accumulation on the fish gill surface has been suggested to be hypoxia [10–11, 15–17].

The crucian carp (*Carassius carassius*) is known to have an extreme ability to survive prolonged anoxia [18–19]. Accordingly, crucian carp are able to survive substantial Al-exposures [20], which is acutely toxic to most other freshwater fish species [21]. It was suggested that this high Al-tolerance in crucian carp might be due to its ability to survive prolonged hypoxia, and even anoxia [20]. However, this association between the ability to survive prolonged hypoxia and Al-tolerance has still to be documented. Moreover, several studies have shown that exposure to dissolved aluminium disrupts ion regulation as well as respiration in fish [10, 22–25]. Therefore, an Al-exposed crucian carp would also have to deal with the negative effects of aluminium on its ion balance, thus there are two different ways that the tolerance to aluminium in crucian carp can be achieved. The aim of this study has therefore been to reveal if the tolerance to aluminium can be associated with the ability to survive prolonged anoxia or not. We have studied the effects of dissolved aluminium during ongoing polymerisation on gill structure, ion regulation and respiration in crucian carp. In light of these results, we will discuss the mechanisms underlying aluminium toxicity in fish in general, and in the crucian carp in particular, focusing on the relationship between the apparent high tolerance to aluminium and the high tolerance to hypoxia/anoxia in crucian carp.

## Materials and methods

### Experimental animals

Crucian carp, ranging from 25 to 80 g in weight and 10 to 15 cm in total length, were obtained from a local pond nearby Oslo, Norway. The fish were caught by basket weir fish traps. No specific permission for trapping was required from the authorities, because it was carried out on private land and did not involve any endangered or protected species. However, permission for trapping was given by the landowner. After trapping, the fish were brought into the fish holding department at the University of Oslo, and kept in dechlorinated Oslo tap water (Table 1) at a flow through of water of 1 l/min. The water was dechlorinated by thiosulphate addition. The fish were fed once a day for at least 2 weeks prior to the experiments. Two days before, and during the experiments, the fish were not fed in order to avoid the influence of food on blood parameters. All animal husbandry conditions and experimental protocols, including sampling procedures and experimental manipulations, reported in this paper were in strict accordance to the guidelines of the Norwegian Animal Research Authority, and approved by the head of the fish holding department at the University of Oslo. Substantial efforts were made to minimize the amount of fish used in the experiments, and to minimize possible suffering during exposures, and to ensure humane endpoint for fish that were sacrificed for sampling.

**Table 1:** Background ion concentrations in the department water during the experimental period.

Ions	Concentrations
Ca^2+^	2.91 ± 0.05 mg/l
K^+^	0.39 ± 0.02 mg/l
Mg^2+^	0.50 ± 0.01 mg/l
Si^2+^	0.98 ± 0.03 mg/l
Na^+^	1.80 ± 0.04 mg/l
F^÷^	72.2 ± 1.47 μg/l
Cl^÷^	2.30 ± 0.04 mg/l
NO_3_^÷^	0.46 ± 0.01 mg/l
SO_4_^2÷^	5.63 ± 0.16 mg/l
Total Fe	25.1 ± 1.03 μg/l
Total Al	50.1 ± 6.87 μg/l

Values are means ± SD (n = 9)

### Test conditions

The experiments were performed in the laboratory of the fish-holding department. Two test media, with a pH of 5.8, were prepared by the addition of various stock solutions to the fish-holding department water: an acidic Al-poor water, by adding a HNO_3_ stock solution (pH 2.0), and an acidic Al-rich water, by adding a Al(NO_3_)_3_ x 9H_2_O plus HNO_3_ stock solution (pH 2.0). Untreated department water (pH 6.5) was used as a reference medium in the experiments.

**Fig 1:**
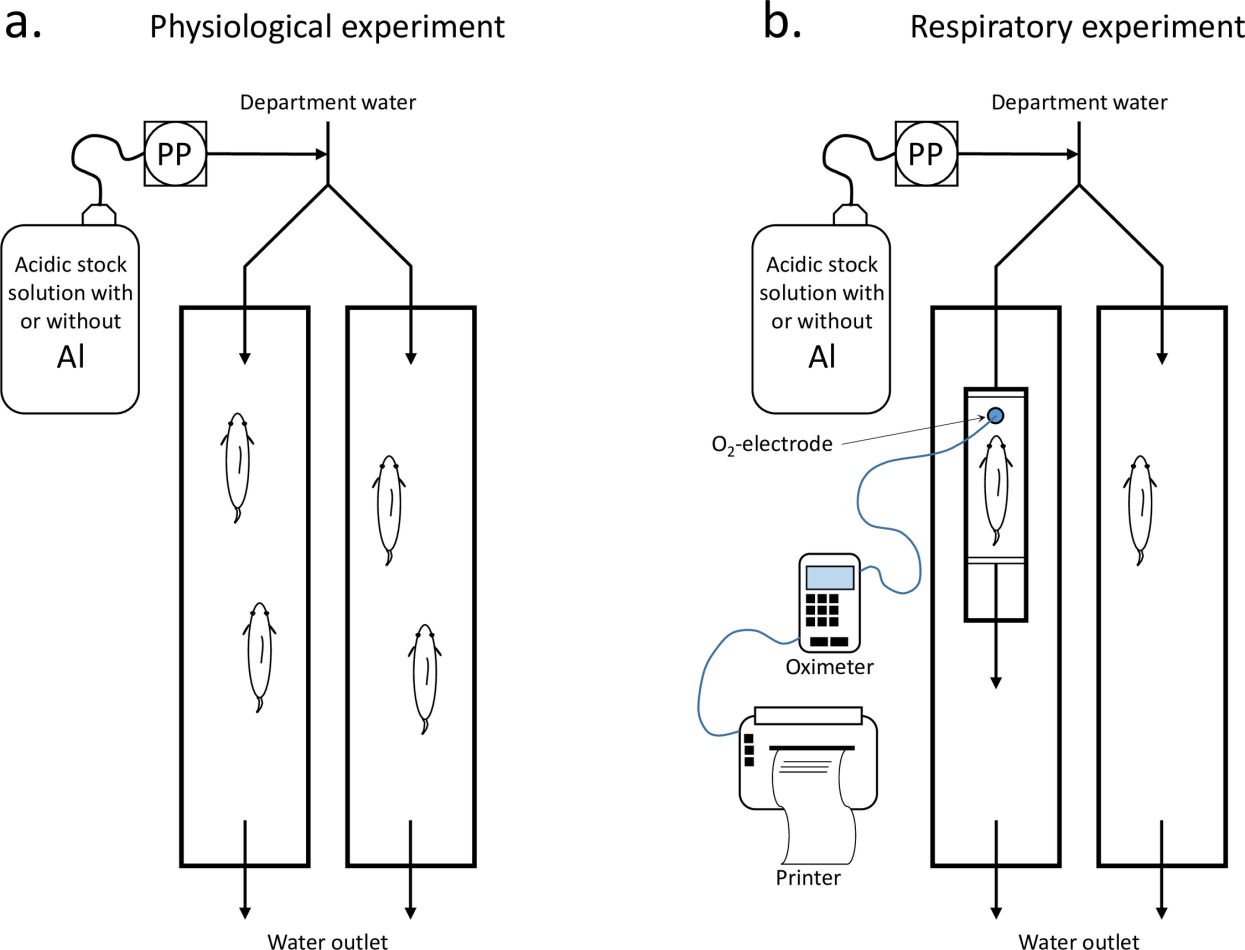
A schematic presentation of the experimental set-ups used in the experiments.

Two different experimental set-ups were made for the experiments. In the first set-up, department water was led through two similar exposure channels (90 cm long, 20 cm wide and 15 cm deep) (Fig 1A). The stock solution for either acidic Al-rich water or acidic Al-poor water were added to the department water as it entered the channels. The second set-up was a modification of the first one, where a respirometer with a volume of 0.7 litres was connected to the water in-let in one of the channels (Fig 1B). Department water was also led through the channel with the respirometer in order to maintain a stable water temperature within the respirometer when it was closed. An O_2_-electrode (WTW CellOx 325) with a magnetic propeller was fitted tightly through a hole at the top of the respirometer. A magnetic stirrer under the channel powered the electrode propeller, securing a homogenous environment within the respirometer. The O_2_-electrode was connected to an amplifier (WTW Oxi 323) which in turn was connected to a printer (W+W 1100).

The water flow-rate through the channels and respirometer was set to approximately 1.5 l/min. The water was well aerated on its way through the channels, and the water flow of 1.5 l/min provided at least 3.0 litres of water per gram fish per day. This is well above 2.0 l/g/day recommended for this kind of experiment [26]. The acidic Al-rich and the acidic Al-poor stock solutions were added to the department water at a rate of approximately 2.5 ml/min using a peristaltic pump. The fish were sheltered from external stress by providing the channels with opaque PVC-covers.

### Experimental protocol

The present study was performed as two separate experiments, referred to as the physiological experiment and respiratory experiment. Both experiments were run as three separate trials, one with the acidic Al-rich water with a nominal concentration of 1000 μg Al/l, one with the acidic Al-poor water, and one with the untreated department water. Water temperature, conductivity and pH were measured daily throughout the experimental periods. The chemical dosage and the water flow through the channels were also controlled each day. During the experiments, aqueous aluminium was analysed at the beginning, the middle and in the end of each experiment. The pH and conductivity measurements were performed immediately after the water samples were collected.

In the physiological experiment, mortality, gill morphology and various physiological parameters; Blood haematocrit, lactate and ethanol concentrations, plasma chloride and sodium concentrations, and muscle water content (MWC) were evaluated. Each trial started when 14 fish were placed in each of the two exposure channels. Prior to this, the stock solution in question had been added to the inlet of the channels for approximately 5 hours. After 2, 6, 10 and 14 days of exposure, 7 individual fish were collected from one of the channels and euthanized with a firm blow to the head, to avoid any effects of anaesthetic drugs and time until fully anaesthetised on the physiological parameters. Subsequently, blood samples were collected from the dorsal artery, and transferred to 50 μl blood caps. 50 μl of blood were stored at -20°C for later analysis of the blood ethanol content, while the remaining blood was centrifuged for 3 minutes at 10000 rpm. Haematocrit was then measured before plasma was isolated and stored at -20°C for later analysis of chloride, sodium and lactate concentrations. The apical gill arch on the left side of the fish was dissected out for later analyses of surface structure by means of Scanning electron microscopy. The gill arches were stored in a protein-fixating buffer (3% glutaraldehyde, 1% formalin, 93% cacodylate buffer; pH 7.4).

In the respiratory experiment the oxygen consumption V_O2_ was monitored by means of closed respirometry, after a previously described protocol [27–28], and the critical oxygen tension [O2]_crit_ was determined as earlier described [29]. 18 fish were used in this experiment, 6 in each trial. Each individual fish were exposed for 6 days to the water in question, before it was transferred to the respirometer. The fish was kept in the respirometer with a continuous flow-through (1.5 l/min) for 15 hours, before the respirometer was closed and the measurements of oxygen uptake started.

### Analytical techniques

pH and temperature were measured using a Radiometer PHM 210 pH-meter. Water conductivity was measured with a Radiometer CDM 80 conductivity meter. The conductivity was determined when three consecutive measurements were identical within one tenth of a unit (μS/cm). Aqueous aluminium was fractionated by the HQ-MIBK extraction technique [30] combined with the cation exchange procedure [31] according to a previously described protocol [21].

The plasma chloride concentration was determined coulometrically using a Radiometer CMT-10 Chloride Titrator, with an expected precision of 0.5%. Plasma sodium concentration was determined by Atomic Absorption Spectrophotometry (AAS). The AAS detection limit was 0.15 ppm. Plasma lactate concentration was measured enzymatically using a Lactate Analyser TDx. Blood ethanol was also analysed enzymatically. Samples of full blood were treated with 6% Perchloric acid (PCA) to separate proteins from the rest of the sample. The sample was then mixed with a Tris-buffer (1 M Tris, 0.3 mM EDTA, 2 mM NAD and 2 μl alcoholdehydrogenase per ml buffer) where all the ethanol reacts with NAD to form Acetaldehyde (bound by Tris) and NADH. The concentration of NADH is equal to that of ethanol and was measured spectrophotometrically at 340 nm. Whole gill arches were postfixed in 1% OsO4, dehydrated in a graded series of ethanol, and critical point dried using CO2. Each gill arch was finally mounted on stubs using epoxy glue, sputter-coated with gold-palladium, and examined in a JEOL 6400 scanning electron microscope. The muscle tissue samples were taken just beneath the dorsal fin on the left side of the fish. The water content of muscle tissue was determined by weighing the samples before and after removal of water in a vacuum freeze dryer (Maxi dry lyo).

All physiological data are presented as means ± standard error of the mean (SEM), while the different fractions of aluminium in the test media are presented as means ± standard deviation (SD). All statistical calculations were carried out using SYSTAT 8.0 (SPSS, 1998). A one-way ANOVA with a Tukey HSD post hoc test for unequal N within each treatment and time point (independent factors) was used to test for a significant effect of the duration of treatment and effect of treatment on the physiological measurements (dependant factors) respectively. A paired t-test was used to examine the difference in oxygen consumption and in critical oxygen tension between fish exposed to acidic Al-poor and Al-rich water and untreated department water.

## Results

### Water chemistry

Water quality parameters and aqueous Al-fractionation data are presented in Tables 2 and 3. In the trials with untreated department water the pH was stable, and remained at 6.5 throughout the experimental periods (Table 2 and S1 Table). In the trials with the acidic waters pH varied between 5.7 and 5.8. The mean water temperature varied between 5.8 and 10.2°C in the physiological experiment, and between 9.5 and 10.2°C in the respiratory experiment. The electrical conductivity of all waters was stable, between 23.7 and 29.6 μS/cm throughout both experiments (Table 2).

**Table 2:** pH, temperature and conductivity in the various waters used in the experiments.

	pH	Temperature (°C)	Conductivity (μS/cm)
**Physiological experiment**			
Acidic Al-rich water	5.8 ± 0.1 (n = 54)	10.2 ± 0.3 (n = 50)	28.0 ± 0.4 (n = 50)
Acidic Al-poor water	5.8 ± 0.1 (n = 48)	5.8 ± 0.5 (n = 48)	24.1 ± 0.5 (n = 48)
Untreated water	6.5 ± 0.1 (n = 48)	7.6 ± 0.5 (n = 48)	24.0 ± 0.4 (n = 48)
**Respiratory experiment**			
Acidic Al-rich water	5.7 ± 0.2 (n = 24)	9.4 ± 0.4 (n = 24)	29.6 ± 2.5 (n = 22)
Acidic Al-poor water	5.7 ± 0.2 (n = 24)	10.5 ± 0.4 (n = 24)	26.7 ± 0.8 (n = 24)
Untreated water	6.5 ± 0.1 (n = 24)	9.0 ± 0.2 (n = 24)	23.7 ± 0.1 (n = 24)

Values are means ± SD. For details, see S1 Table.

**Table 3:** Al-concentrations (μg/l) in the various waters used in the experiments.

		Acidic Al-rich water	Acidic Al-poor water	Untreated water
Physiological experiment				
Alr	inlet	929 ± 250 (n = 5)	44 ± 5 (n = 5)	47 ± 4 (n = 5)
	outlet	656 ± 136 (n = 5)	38 ± 4 (n = 5)	43 ± 6 (n = 5)
Ala	inlet	400 ± 41 (n = 6)	21 ± 4 (n = 5)	23 ± 6 (n = 5)
	outlet	416 ± 56 (n = 6)	16 ± 4 (n = 5)	23 ± 6 (n = 5)
Alo	inlet	148 ± 15 (n = 6)	11 ± 6 (n = 5)	11 ± 4 (n = 5)
	outlet	165 ± 29 (n = 6)	10 ± 4 (n = 5)	16 ± 4 (n = 5)
Ali	inlet	252 ± 50 (n = 6)	10 ± 5 (n = 5)	11 ± 7 (n = 5)
	outlet	251 ± 78 (n = 6)	7 ± 4 (n = 5)	2 ± 3 (n = 5)
Respiratory experiment				
Alr	inlet	878 ± 160 (n = 3)	46 ± 6 (n = 3)	56 ± 7 (n = 3)
	outlet	666 ± 89 (n = 3)	47 ± 4 (n = 3)	52 ± 5 (n = 3)
Ala	inlet	673 ± 223 (n = 3)	22 ± 7 (n = 3)	25 ± 7 (n = 3)
	outlet	469 ± 107 (n = 3)	17 ± 7 (n = 3)	25 ± 8 (n = 3)
Alo	inlet	155 ± 23 (n = 3)	11 ± 6 (n = 3)	18 ± 5 (n = 3)
	outlet	160 ± 18 (n = 3)	12 ± 10 (n = 3)	20 ± 7 (n = 3)
Ali	inlet	518 ± 230 (n = 3)	11 ± 4 (n = 3)	7 ± 4 (n = 3)
	outlet	309 ± 123 (n = 3)	6 ± 3 (n = 3)	5 ± 3 (n = 3)

Values are means ± SD. Water samples for Al-analyses were collected at the inlet and outlet of each channel during the different exposures.

During the physiological experiment the total aluminium concentration (Alr) in the Al-rich water was 929 ± 250 μg/l at the inlet, and 656 ± 136 μg/l at the outlet of the exposure channels. At the inlet approximately 43% of this amount was present as monomeric Al-species (Ala: 400 ± 41 μg/l), and approximately constituted 63% (Ala: 416 ± 56 μg/l) at the outlet (Table 3 and S2 Table). The calculated inorganic monomeric Al-fraction (Ali) was 252 ± 50 μg/l (63% of the Ala-fraction) at the inlet, and 251 ± 78 μg/l (60% of the Ala-fraction) at the outlet of the exposure channels. In the acidic Al-poor and the untreated waters, the Alr-concentrations varied between 38 and 47 μg/l. The Ala fraction varied between 16 and 23 μg/l, while the Ali fraction varied between 1 and 11 μg/l.

During the respiratory experiment the Alr concentration in the Al-rich water was 878 ± 160 μg/l at the inlet, and 666 ± 89 μg/l at the outlet of the exposure channels. At the inlet, approximately 77% of this amount was present as Ala (673 ± 223 μg/l), and approximately 70% (Ala: 469 ± 107 μg/l) at the outlet (Table 3 and S2 Table). The calculated Ali-fraction was 518 ± 230 μg/l (77% of the Ala-fraction) at the inlet, and 309 ± 123 μg/l (66% of the Ala-fraction) at the outlet. In the acidic Al-poor and the untreated waters, the Alr-concentrations varied between 46 and 56 μg/l. The Ala fraction varied between 17 and 25 μg/l, while the Ali fraction varied between 5 and 11 μg/l.

### Al-polymerisation

The water chemistry analyses show that the experimental fish was exposed to an ongoing polymerisation of aluminium in the trials with acidic Al-rich water. This is supported by the observed decrease in the total amount of dissolved aluminium (Alr-fraction) as the water moved through the experimental set-up. During polymerisation, the Al-compounds will increase in size, and eventually fall out of solution and hence become undetectable [32–33]. Moreover, we also observed that the Alo-fraction was larger in the acidic Al-rich water compared to the acidic Al-poor water. The Alo-fraction is operationally defined as organic monomeric Al-species [31]. However, the concentration of total organic compounds (TOC) in the department water is below 3 mg C/l [21] and should be the same in the two acidic waters. Therefore, the larger concentration of Alo is most probably caused by Al-polymerisation. Because large Al-polymers have weak, or no net electrical charge, they pass through the cation exchanger [31] and are extracted from the solution [30], and will be detected as organic aluminium even though they are inorganic [33].

### Mortality

There was no mortality observed among fish exposed to aluminium during the experimental periods. In general, however, we observed a change in behaviour in fish exposed to aluminium. The fish showed a decreasing swimming activity compared to the control fish, and there was less response to handling prior to sampling.

### Blood and plasma parameters

**Fig 2:**
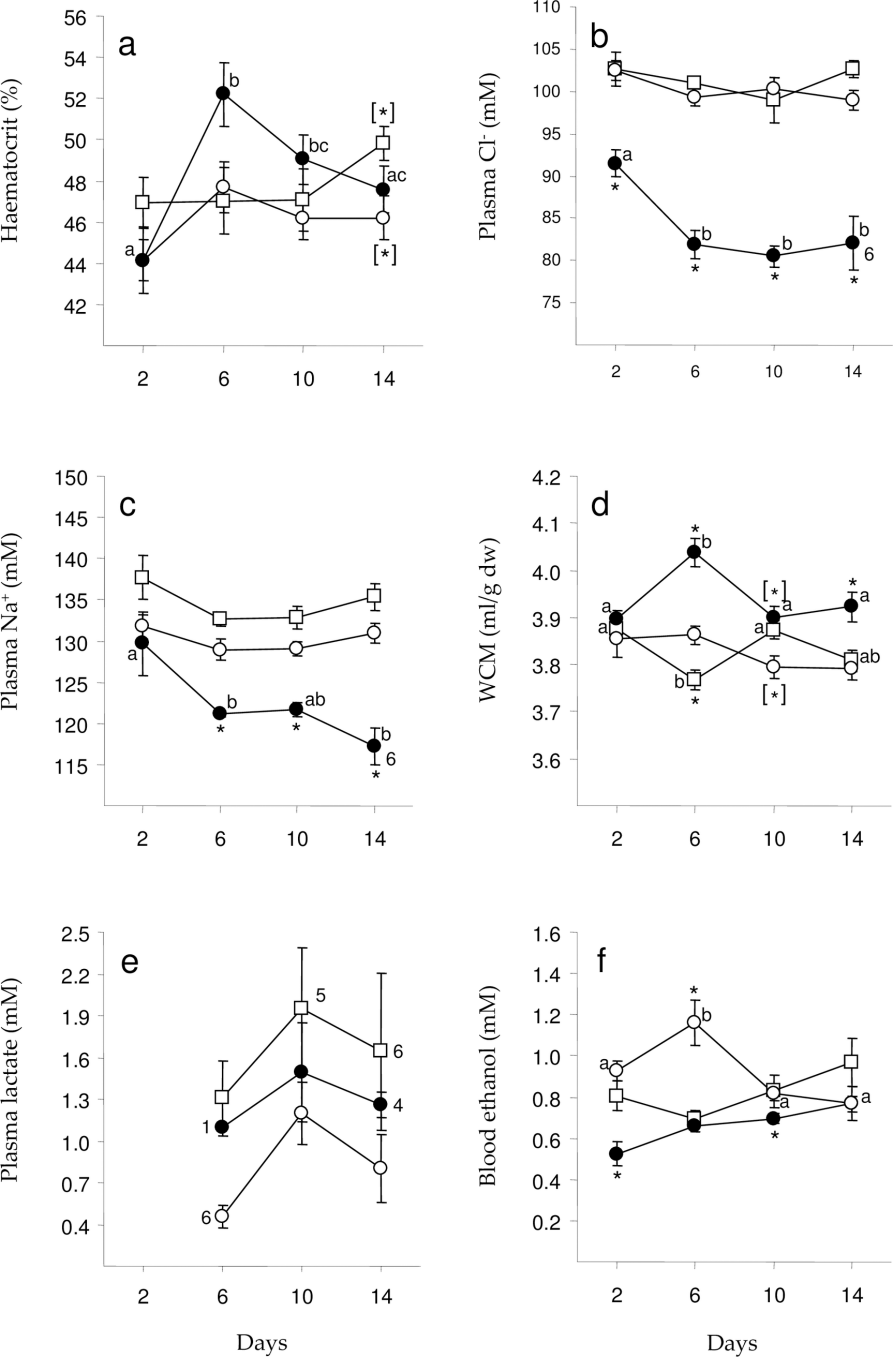
Blood and plasma parameters. Mean values (± SEM) of haematocrit (a), plasma chloride (b), plasma sodium, (c), water content in muscle tissue (d), plasma lactate (e), and blood ethanol (f) in crucian carp after 2, 6, 10 and 14 days of exposure to the acidic Al-rich water (closed circles), acidic Al-poor water (open circles) and untreated department water (open squares). n = 7 if not noted differently on the graph. Time points within experimental groups assigned with different letters are significantly different from each other (p < 0.05). Experimental groups assigned with asterisks (*) displayed significant differences (p<0.05) in comparison to the other two groups. Experimental groups assigned with asterisks in brackets ([*]) displayed a significant difference in physiological parameters in comparison to only one of the other groups (also assigned with [*]). All data are listed in S3 Table.

The analyses of blood and plasma parameters revealed some negative effects on the physiology in fish exposed to the acidic Al-rich medium (Fig 2).

Haematocrit in fish exposed to the untreated and the acidic Al-poor water did not change significantly (F_3,24_ = 1.094, p = 0.371 and F_3,24_ = 1.208, p = 0.328 respectively) during the trials (Fig 2A) and remained between 44.1 ± 1.6% and 49.8 ± 0.8%. However, there was a significant effect of duration of exposure in fish from the acidic Al-rich water (F_3,24_ = 7.441, p = 0.001), where haematocrit increased from 44.1 ± 1.0% to 52.2 ± 1.5% between day 2 and 6 (p = 0.001), but not enough to become significantly higher compared to the other groups sampled at day 6 (p = 0.056). Between day 6 and 10 haematocrit decreased significantly (Fig 2A) in fish exposed to the acidic Al-rich medium (p = 0.035), and ended up at 47.6 ± 1.1% on day 14, not significantly different from the two other groups (p > 0.220). Moreover, haematocrit did not differ between fish exposed to untreated and acidic Al-poor water (p > 0.959) except on day 14 when haematocrit in fish in the untreated water was significantly higher compared to the other sampling days (p = 0.041).

Plasma chloride in fish exposed to untreated and acidic Al-poor water did not change significantly (F_3,24_ < 1.778, p > 0.178) during the trials (Fig 2B), and remained between 99 ± 3 and 103 ± 1 mM. However, there was a significant effect of exposure in fish from the trial with acidic Al-rich water (F_3,23_ = 6.172, p = 0.003), where plasma chloride decreased from 91 ± 2 to 82 ± 2 mM between day 2 and 6 (p = 0.014). Plasma chloride did not decrease any further after day 6 (p > 0.947), and remained lower compared to day 2 throughout the exposure (p < 0.022). On all days plasma chloride in Al-exposed fish was significantly (F_2,18_ > 14.557, p < 0.001) lower compared to the other two groups, which did not differ significantly (p > 0.622) at any time during the experiment.

Plasma sodium in fish exposed to untreated and acidic Al-poor water did not change significantly (F_3,24_ = 1.991, p = 0.142 and F_3,23_ = 0.962, p = 0.427 respectively) during the trials, and remained between 129 ± 1 and 137 ± 3 mM. (Fig 2C). However, there was a significant effect of duration of exposure in fish from the trial with acidic Al-rich water (F_3,23_ = 5.185, p = 0.007), where plasma sodium was significantly lower at day 6 (121 ± 1 mM) (p = 0.050) and day 14 (117 ± 2 mM) (p = 0.005) compared to day 2 (129 ± 4 mM). However, there were no significant changes in plasma sodium in the period from day 6 to day 14 (p > 0.693). Except for day 2 (F_2,18_ > 2.050, p = 0.158), plasma sodium in Al-exposed fish was significantly (F_2,17_ > 25.010, p < 0.001) lower compared to the other two groups. Although plasma sodium in fish exposed to the acidic Al-poor water was consequently lower compared to fish exposed to the two other waters, there were no significant (p > 0.057) differences between these two groups at any time during the trials.

The muscle water content (MWC) in fish exposed to the acidic Al-poor water did not change significantly (F_3,24_ = 1.911, p = 0.155) during the trial, and remained between 3.79 ± 0.02 and 3.86 ± 0.02 ml/g dry weight (dw) (Fig 2D). However, there was a significant effect of duration of exposure in fish from the trial with untreated water as well as fish from the acidic Al-rich water (F_3,24_ = 5.757, p = 0.004 and F_3,24_ = 6.442, p = 0.002 respectively). MWC in fish from the untreated water was significantly lower at day 6 (3.76 ± 0.02 ml/g dw) compared to day 2 and 10 (3.87 ± 0.02; p = 0.010 for both comparisons). In Al-exposed fish MWC was significantly higher at day 6 (4.04 ± 0.03 ml/g dw) compared to day 2, 10 and 14 (day 14 highest with 3.92 ± 0.03 ml/g dw) (p < 0.022). There was also a significant difference between the groups at day 6, 10 and 14 (F_2,18_ > 6.374, p < 0.008). At day 6, MWC was higher in Al-exposed fish compared to the other two groups (p < 0.001), while fish exposed to the acidic Al-poor water had higher MWC compared to the fish from the untreated water (p = 0.036). After 10 days of exposure the Al-exposed fish had higher MWC than fish exposed to the acidic Al-poor water (p < 0.008), but neither of these groups differed from the fish from the untreated water (p > 0.051). At day 14, Al-exposed fish had higher MWC compared to the other two groups (p < 0.018), whereas these did not differ (p = 0.890).

Plasma lactate did not change significantly in any of the trials (F_3,23_ < 1.992, p > 0.154) during the exposures, and remained between 0.5 ± 0.1 and 1.6 ± 0.6 mM (Fig 2E). Moreover, there were no significant differences in plasma lactate between any of the groups (F_2,11_ > 3.525, p < 0.066) during the experiment.

Blood ethanol in fish exposed to untreated and acidic Al-rich water did not change significantly (F_3,24_ = 1.963, p = 0.151 and F_3,24_ = 1.963, p = 0.151 respectively) during the trials, and remained between 0.69 ± 0.04 and 0.96 ± 0.11 mM in fish from the untreated water, and between 0.52 ± 0.06 and 0.77 ± 0.08 mM in Al-exposed fish (Fig 2F). However, there was a significant effect of duration of exposure in fish from the Al-poor water (F_3,23_ = 4.726, p = 0.010), where blood ethanol increased significantly between day 2 (0.92 ± 0.05 mM) and 6 (1.16 ± 0.11 mM) (p = 0.038), and decreased between day 6 and 14 (0.76 ± 0.04 mM) (p = 0,011). There was a significant difference between the groups on day 2, 6 and 10 (F_2,18_ > 9.252, p < 0.002). Blood ethanol in Al-exposed fish was significantly lower compared to the other two groups on day 2 (p < 0.003) and 10 (p < 0.018), and lower in Al-exposed fish and fish from the untreated water compared to fish from the Al-poor water on day 6 (p < 0.008).

### Gill morphology

**Fig 3:**
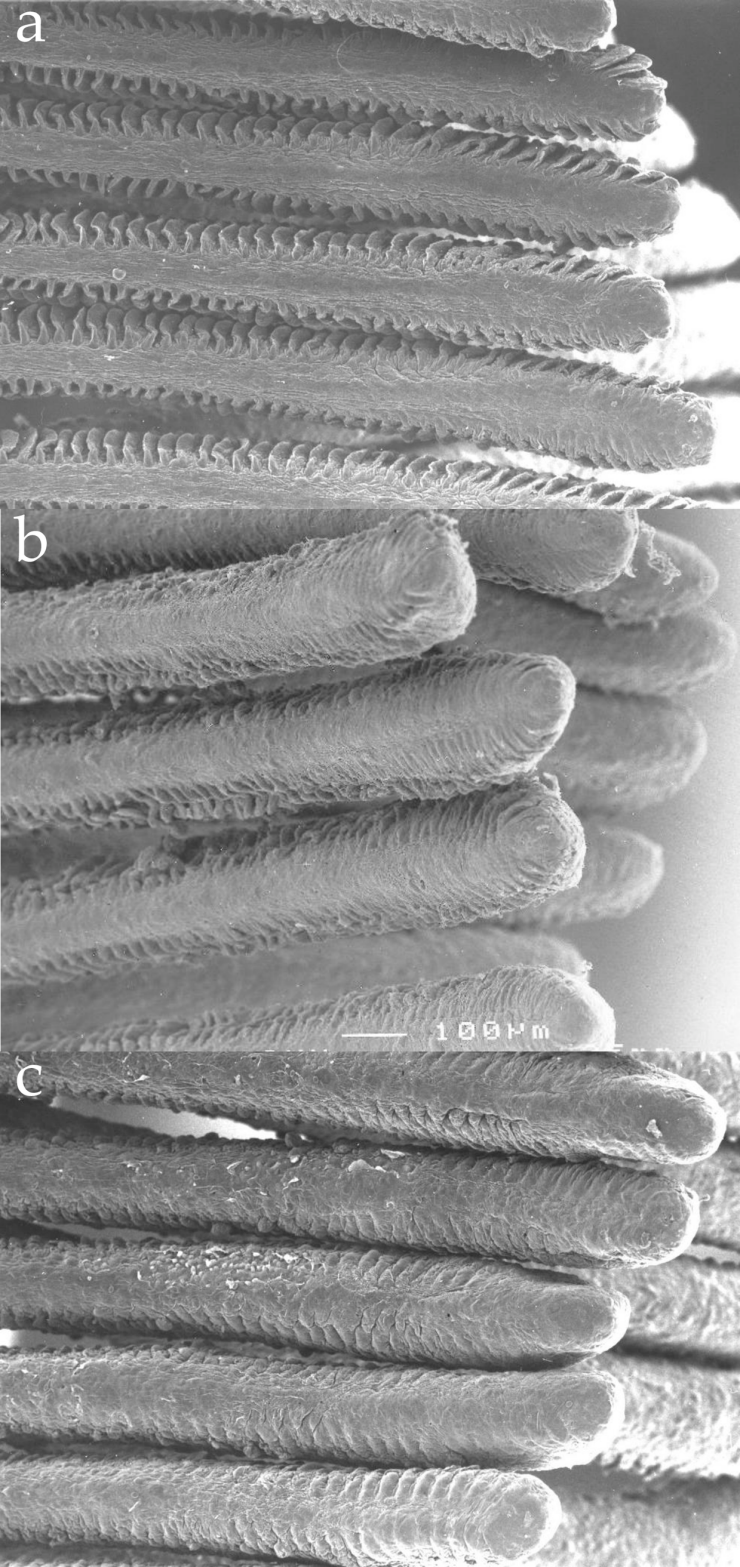
**Scanning electron micrographs** of gill filaments from the apical right side of crucian carp, exposed to acidic Al-rich water (a), acidic Al-poor water (b), and untreated department water (c).

The gills from fish exposed to the acidic Al-rich water showed no sign of structural damage (Fig 3). However, the lamellar surface of the gills from fish exposed to the acidic Al-rich water was larger, with clearly visible secondary lamellae present, compared to fish exposed to untreated and acidic Al-poor water, where secondary lamellae were not clearly visible (Fig 3). The gills from fish exposed to the acidic Al-poor water showed a similar structural morphology as the gills from fish in the untreated water.

### Oxygen uptake

Fish exposed to acidic Al-rich water showed a normoxic O_2_-consuption at 30 ± 4 mg/kg·h O_2_ (n = 6). In comparison, the normoxic O_2_-consuption in fish exposed to untreated and acidic Al-poor water was 54 ± 15 and 63 ± 11 mg/kg·h O_2_ (n = 6) respectively (Table 4). There were no significant differences between fish exposed to untreated water compared to fish from the acidic Al-poor water (F_1,10_ = 0.208, p = 0.658), or compared to fish from the acidic Al-rich water (F_1,10_ = 2.407, p = 0.151). However, there was a significant difference between fish exposed to the acidic Al-poor water compared to fish from acidic Al-rich water (F_1,10_ = 8.380, p = 0.016). Moreover, there were no significant differences in critical O_2_-tension between fish exposed to the different waters (F_1,10_ < 2.435, p > 0.150). Critical O_2_-tension was 1.9 ± 0.2 mg/l O_2_ in fish exposed to acidic Al-rich water, 1.4 ± 0.2 mg/l O_2_ in fish exposed to acidic Al-poor water and 1.6 ± 0.3 mg/l O_2_ in fish exposed to untreated water.

**Table 4:** Oxygen consumption (V_O2_) and critical oxygen tension ([O_2_]_crit_) in crucian carp exposed to various waters.

	Respiratory measurements	
Groups	V_O2_ (mg O_2_/kg•h)	[O_2_]_crit_ (mg O_2_/l)
Acidic Al-rich water	30 ± 4*	1.9 ± 0.2
Acidic Al-poor water	63 ± 11*	1.4 ± 0.2
Untreated department water	54 ± 15	1.6 ± 0.3

Values are means ± SEM of six fish. Asterisks denote significant difference between the groups (p < 0.01). All measurements are listed in S4 Table.

## Discussion

The results from the present study confirm that crucian carp survives exposure to substantial amounts of toxic aluminium. However, the exposure to aluminium during ongoing polymerisation did have an effect on the physiology of the crucian carp, and it was different from previous observations in other fish species exposed to aluminium. Our results indicate that the high tolerance to aluminium in crucian carp is associated with its ability to live under hypoxic or anoxic conditions, and a capacity to counteract the loss of plasma ions to the surrounding environment.

### Acute Al-toxicity

In agreement with a previous study [20], our results show that crucian carp has a high tolerance to aluminium. In fact, the present study has documented that crucian carp can survive exposures to more than twice the concentrations of aluminium, approximately 970 μg/l, compared to approximately 400 μg/l used in an earlier study [20]. In the present study crucian carp was also exposed to a larger degree of ongoing Al-polymerisation than previously [20], conditions in which Al-toxicity in fish is most severe [10, 16]. The toxicity of aluminium seems to be dependent on the concentration of positively charged inorganic monomeric species, and their interaction with the negatively charged gill surface [3, 34]. In the present study, crucian carp was exposed to 385 μg Al_i_/l. To our knowledge, no other freshwater fish species have been able to survive exposures to similar Al_i_-concentrations. It has been demonstrated that aluminium was acutely toxic to seven common Scandinavian freshwater fish species at 300 μg Al_i_/l [21]. Our results therefore document that crucian carp can survive Al-exposures substantially more severe than Al-exposures shown to be acutely toxic to other fish species.

### Effects of aluminium on gill structure

Based on the high concentration of positively charged inorganic monomeric Al-species present in our Al-exposure, we would expect an interaction between these Al-species and the crucian carp gills. It has been demonstrated that Al-exposed crucian carp accumulated substantial amounts of aluminium on their gills [20]. We did not measure the amount of aluminium on the fish gills, but our results revealed no signs of structural damage to the gill surface, even though the conditions for Al-polymerisation and accumulation on the fish gills were more extensive in the present study compared to the earlier study [20]. Furthermore, our results are in strong contrast to previous studies on the effects of aluminium on gill structure in other fish species [16, 35–37]. Consequently, the absence of gill damage suggests that crucian carp, to some extent, has the ability to avoid the effects of Al-toxicity. In fact, our results showed that the area of the gill surface was increased as a consequence of enlarged lamellae in Al-exposed crucian carp compared to controls. This might be one way in which crucian carp copes with the consequences of Al-induced hypoxia. It is worth mentioning at this point that the gill morphology of crucian carp is deviating from what is normal in other fish species. Normally, fish has thin primary lamellae with large protruding secondary lamellae [38–40]. Our results are therefore in agreement to earlier findings [27] that crucian carp lacked protruding lamellae when maintained in clean and normoxic water, while the lamellar surface increased more than seven-fold when the fish were transferred to a hypoxic environment for seven days. This phenomenon was reversible when reintroduced to normoxic water, and shows that crucian carp has the ability of an adaptive morphological change in gill structure as a response to a hypoxic environment [27], or hypoxia induced by accumulation of aluminium on the gill surface, as indicated by the present study and an earlier study [20].

### Effects of aluminium on respiration

It is well documented that respiration is affected in fish exposed to acidic Al-rich water [3, 22]. Our results indicate that this is not the case in crucian carp. Al-exposed crucian carp showed a significantly lower O_2_-uptake compared to fish exposed to the acidic Al-poor water. It should be noted, however, that our results on O_2_-uptake are inconclusive, because the O_2_-uptake was not significantly lower in Al-exposed fish compared to fish exposed to untreated department water. On the other hand, the lack of a continued elevation in haematocrit, plasma lactate and blood ethanol, support the opinion that Al-exposed crucian carp did not become hypoxic. Moreover, the present study documents that the critical oxygen tension for crucian carp did not increase as a consequence of exposure to aluminium, indicating that O_2_-consumption was not restricted. Overall, our results speak in favour of a conclusion that crucian carp exposed to acid Al-rich water does not become hypoxic, as do other fish species [3, 22].

The increased gill surface area and the reduced O_2_-uptake in Al-exposed crucian carp do not agree with the statement that crucian carp exposed to aluminium does not become hypoxic. This contradiction can be explained by a reduction in metabolic rate. It has been documented that goldfish (*Carassius auratus*), a close relative to crucian carp, can reduce its metabolism during anoxia by 70% [41]. Moreover, it has been shown that crucian carp reduces its energy expenditure during hypoxic/anoxic conditions [42], and it was found that this was achieved by reducing the spontaneous locomotor activity during anoxia [43]. When fish are exposed to ongoing Al-polymerisation, aluminium will accumulate on the gills [10, 16, 44], leading to an increased secretion of mucus on the gill surface [35, 45–46]. Consequently, the diffusion barrier for oxygen will increase [47], resulting in local hypoxia at the gill surface, which can be detected by the external O_2_-receptors in the gills. A local hypoxia at the gill surface can serve as a stimulus for energy saving strategies, as well an increased gill surface area, to cope with the hypoxic/anoxic challenge [42–43]. In addition, this can explain the lower O_2_-uptake combined with the absence of hypoxic indicators we observed in Al-exposed fish. Overall, the results from the present study support the suggestion that the crucian carp is able to meet its O_2_-demand when exposed to an Al-challenge that would lead to acute asphyxiation in other freshwater fish species. In addition, we suggest that the increased gill surface area is the reason why the critical O_2_-tension did not increase in Al-exposed fish.

### Effects of aluminium on ion regulation

Increasing the gill surface as a response to hypoxia/anoxia will have an effect on the osmo-respiratory compromise [48]. Hence, the present study documents that ion regulation is affected in crucian carp exposed to high concentrations of toxic aluminium. In contrast to previous studies on fish showing that the levels of plasma ions decrease continuously during Al-exposures [25, 45], our results show that both plasma chloride and sodium stabilises at a lower level, compared to control, after an initial decrease. This is in agreement with previous results obtained from Al-exposed crucian carp [20]. It is generally agreed that the decrease in plasma ions in Al-exposed fish is caused by an increased passive efflux across the gill epithelium, as well as a reduction in the active uptake of ions from the surrounding water [10, 23, 49–50]. The levelling off of the ion loss that we observed, could be a result of an ability in crucian carp to increase the active uptake of ions, or caused by a decreasing diffusion gradient between blood and the surrounding water as the plasma ion concentrations decrease. We have previously suggested this as an explanation for reduced ion loss rate in copper exposed crucian carp [28]. The ion transport across the gill epithelium is an energy demanding process, and since the normoxic O_2_-uptake in crucian carp exposed to aluminium did not exceed that of control fish, it seems unlikely that the compensation of ion loss is achieved by an increase in ion transportation, which has been suggested as a mechanism for counteracting ion loss in other fish species during Al-exposure [51]. It therefore seems more likely that the decreased ion loss rate in Al-exposed crucian carp is a result of a reduced diffusion gradient across the gill epithelium. Since the levels of plasma ions stabilise in crucian carp, as opposed to other fish species, the passive efflux of plasma ions seems to have come into equilibrium with the active uptake. This can be achieved because crucian carp has a small gill surface area compared to other fish species [27], despite that the gill surface area in Al-exposed crucian carp increased somewhat. The small gill surface in crucian carp compared to other fish species might be the reason why equilibrium between passive efflux and active uptake is obtained [27]. In their experiment, crucian carp with no protruding lamellae showed lower plasma chloride concentrations when exposed to a salinity of 16 ppm compared to fish with protruding lamellae.

In our study, the initial reduction in plasma ion concentrations in Al-exposed crucian carp seems to be followed by a short transient increase in haematocrit and muscle water content (MWC) (Fig 2). These transient changes correspond with the reduced ion loss rate observed. An increase in haematocrit could be caused, partly by reduced blood volume, partly by swelling of red blood cells or by release of red blood cells from the spleen. Since the crucian carp in our study did not become hypoxic it seems likely that the transient increase in haematocrit was caused by an increased volume of red blood cells combined with a reduction in plasma volume rather than an increase in the number of red blood cells. This is supported by the corresponding transient increase in MWC. Thus, the decrease in both haematocrit and MWC, back to control levels when the net efflux of ions ceased, suggests that the crucian carp were able to recover from a transient volume regulatory disturbance. This suggestion is supported by an earlier study reporting no change in haematocrit in Al-exposed crucian carp [20]. The observed ability of Al-exposed crucian carp to prevent a continuous loss of plasma ions, and subsequently regulate its ion and water balance, seems to contribute significantly to its high tolerance to toxic aluminium. This is supported by a resent study showing that mortality in copper exposed crucian carp did not occur before levels of plasma chloride had decreased to 40% of control levels [28]. In the present study, and an earlier study [20], the plasma chloride reduction in Al-exposed crucian carp levelled off at approximately 80% of control levels.

The water temperature in the trial with the Al-rich water was somewhat higher, 10.2°C in average, than in the two other trials, between 5.8 and 7.6°C in average. It can be argued that this could be the reason why Al-exposed fish responded different from the fish in the two other groups. It has been demonstrated earlier, however, that Atlantic salmon (*Salmo salar*) exposed to different temperatures between 1 and 19°C in an Al-poor medium did not show any significant difference in blood haematocrit, plasma osmolality and plasma concentrations of sodium and chloride [45]. Taken into account that the temperature difference in the present study was minor compared to the previous study on Atlantic salmon, and the fact that the Atlantic salmon is far more sensitive to acidified waters than the crucian carp [20–21], we believe that the differences in physiological response observed in the present study should be explained by the Al-exposure only.

## Conclusion

Our study has shown that crucian carp is able to survive exposures to much higher concentrations of positively charged low molecular weight Al-species, and more favourable conditions for Al-polymerisation, than previously shown to be acutely toxic to other fish species. The gills of Al-exposed crucian carp show no signs of structural damage and there were no increase in the critical oxygen tension as a consequence of Al-exposure. Moreover, only small and transient changes in haematocrit, plasma lactate, and blood ethanol indicated that the aluminium did not cause hypoxia in crucian carp. However, the oxygen uptake was reduced in Al-exposed crucian carp, which could be assigned to a lowering of metabolism due to local hypoxia at the gill surface. Since the crucian carp in our study did not become hypoxic, we cannot conclude that the high tolerance to toxic aluminium must be acquired by its hypoxia/anoxia tolerance. In our particular case, it seems that the crucian carp is able to survive exposures to toxic aluminium because it avoids hypoxia. Furthermore, Al-exposure caused a net loss of plasma chloride and sodium in crucian carp. However, our results on ion regulation are exceptional because crucian carp was able to counteract a continuous loss of plasma ions and re-establish the water-ion balance in blood and muscle tissue. This indicates that the net loss of plasma ions was reduced due to a reduction in the passive efflux to the surrounding environment. However, we cannot exclude that the stabilisation of plasma ion concentrations in Al-exposed crucian carp was caused by an increase of the active transport of ions. On the other hand, an increase in active transport of ions acquire energy, and does not fit with the fact that Al-exposed crucian carp showed a lower O_2_-uptake compared to controls. Anyhow, the ability to counteract the loss of ions plays a major role in the high tolerance to toxic aluminium, especially since the crucian carp does not become hypoxic. The fact that the crucian carp seems to avoid hypoxia and shows only minor symptoms of Al-toxicity agrees with the hypothesis that hypoxia is the primary effect of Al-exposure in fish, especially when conditions are in favour of Al-polymerisation and accumulation on the gill surface [10].

## Supporting information

S1 Table Water pH, temperature and conductivity measurements. (XLS)

S2 Table Aluminium fractionations. (XLS)

S3 Table Physiological measurements from individual fish in the physiology experiment. (XLS)

S4 Table Physiological measurements from individual fish in the respiratory experiment. (XLS)
